# Universal relation between spectral and wavefunction properties at criticality

**DOI:** 10.1073/pnas.2518027123

**Published:** 2026-02-06

**Authors:** Simon Jiricek, Miroslav Hopjan, Vladimir Kravtsov, Boris Altshuler, Lev Vidmar

**Affiliations:** ^a^Department of Physics, Faculty of Mathematics and Physics, University of Ljubljana, Ljubljana SI-1000, Slovenia; ^b^Department of Theoretical Physics, J. Stefan Institute, Ljubljana SI-1000, Slovenia; ^c^Institute of Theoretical Physics, Wrocław University of Science and Technology, Wrocław 50-370, Poland; ^d^International Center for Theoretical Physics, Trieste 34151, Italy; ^e^Department of Physics, Columbia University, New York, NY 10027

**Keywords:** critical behavior, spectral statistics, wavefunction multifractality

## Abstract

Quantum-chaotic systems exhibit several universal properties, ranging from level repulsion in the energy spectrum to wavefunction delocalization. On the other hand, if wavefunctions are localized, the energy levels exhibit no level repulsion and their statistics is Poisson. At the boundary between quantum chaos and localization, however, one observes critical behavior, not complying with any of those characteristics. An outstanding open question is whether there exists yet another type of universality, which is genuine for the critical point. Previous work suggested that there may exist a relation between the global characteristics of the energy spectrum, such as the spectral compressibility *χ*, and the degree of wavefunction delocalization, expressed via the fractal dimension $D_{1}$ of the Shannon–von Neumann entropy in a preferred (e.g., real-space) basis. Here, we study physical systems subject to local and nonlocal hopping, both with and without time-reversal symmetry, with the Anderson models in dimensions three to five being representatives of the first class, and the random banded matrices as representatives of the second class. Our thorough numerical analysis supports the validity of the simple relation $\chi +D_{1}=1$ in all systems under investigation. Hence, we conjecture that it represents a universal property of a broad class of critical models. Moreover, we test and confirm the accuracy of our surmise for a closed-form expression of the spectral compressibility in the one-parameter critical manifold of random banded matrices. Based on these findings, we derive a universal function $D_{1}\left(r\right)$ of the averaged level spacing ratio *r*, which is valid for a broad class of critical systems.

## Significance

An important role in physics research is to uncover universal properties of various systems with different microscopic descriptions. Examples of microscopic models that exhibit paradigmatic properties are those that describe chaotic quantum dynamics and have spectral and wavefunction properties governed by random-matrix theory. Radical counterexamples to this behavior are also known, and one of such cases is the well-known Anderson localization. Nevertheless, much less is known about the possible universal properties at the boundary between quantum chaos and localization. Here, we conjecture and confirm, using large-scale numerical simulations, a universal relation between the spectral compressibility and the wavefunctions’ fractal dimension at criticality. This result paves way toward searching analogous relations in interacting models.

Quantum-chaotic systems are generic quantum systems characterized by eigenlevels that follow Wigner-Dyson random-matrix statistics (1) and by eigenstates that are delocalized in essentially any basis. Notable examples of such systems are complex nuclei (2), quantum dots (3), quantum billiards (4), weakly disordered electrons in high-dimensional lattices (5), and interacting many-body systems (6). Oppositely, localized systems exhibit Poisson eigenlevel statistics, which are drastically different from Wigner-Dyson level statistics, since the former correspond to the absence of level repulsion (1). Eigenstate statistics in such systems are basis-dependent, with localization occurring in some preferred basis. In case of electrons propagating in lattices with large on-site disorder, the preferred basis is the site-occupation basis.

Here, we focus on the critical boundary between these two classes of systems. Critical systems are associated with intermediate eigenspectra statistics, i.e., neither of Wigner-Dyson nor Poisson type, and with multifractal eigenstates, i.e., neither delocalized over the entire Hilbert space nor localized, suggesting that the change in the eigenlevel spectrum is connected to the change in the extension of the eigenstates.

Early studies of the critical eigenspectra were mostly focused on the level statistics at the critical point of the localization transition in the three-dimensional (3*d*) Anderson model, specifically, on the level number variance in a certain spectral window (7) and the level spacing distribution function (8). These works showed that the critical level statistics exhibit a hybrid character. With the Poisson statistics, they share the linear dependence of the level number variance as a function of the mean number of levels in the window, but also the simple exponential decay of the level spacing distribution at large gaps between levels. However, at small gaps the critical statistics exhibit level repulsion, similar to the Wigner-Dyson case.

The next important step concerned the critical eigenfunction statistics, inspired by the discovery of multifractality by F. Wegner (9). It was shown that the critical eigenfunctions are characterized by a set of fractal dimensions $0<D_{q}<1$, which are intermediate between the case of fully extended (ergodic) wavefunctions, where all $D_{q}=1$, and the case of localized wavefunctions, where all $D_{q}=0$ (10).

That said, not all $D_{q}$ are equally important for the description of critical eigenfunctions. For physical applications related to pair interaction, e.g., for multifractal superconductivity near the localization transition (11), the most relevant is the fractal dimension $D_{2}$. However, the principal information about the fractal character of a wavefunction is encoded in the fractal dimension $D_{1}$; see also the discussion below.

At criticality, a nontrivial question emerges about a quantitative relationship between the eigenvalue statistics and the eigenfunctions’ fractal dimensions. On first glance this question is not legitimate, since the spectrum is basis-invariant while the eigenfunctions are not. Yet, our experience shows that this relation may exist for physically motivated models. An evidence is the observation, which has become common wisdom, that Poisson spectral statistics correspond to localized wavefunctions and Wigner-Dyson statistics correspond to fully ergodic states. Hence, there must exist a basis (or a manifold of bases), denoted as the preferred basis, in which localization effects are strongest, corresponding to the minimal fractal dimension $D_{1}$. In most studies of Anderson localization, the preferred basis coincides with the “natural” one in which the model is formulated, e.g., by locality of disorder potential in this basis.

We note that the preferred basis should be fixed, i.e., independent of a specific realization in a given ensemble of random Hamiltonians. Thus the basis of eigenstates, in which all states are trivially localized, should be excluded, as it depends on the particular realization of disorder. The states are called “localized” when $D_{1}=0$ in the preferred basis.

## Conjecture About the Universal Relation at Criticality

The above discussion suggests that when compared to the spectral properties, the wavefunction properties should be expressed in the preferred basis. The goal of this paper is to establish a link between the wavefunction and spectral properties at the localization transition in physical models. Finding their universal relations, whenever they exist, is important since they may establish a hallmark of critical behavior in large but finite systems.

The first observation of a possible relation between the spectral and the eigenfunction properties was suggested in 1996 by Chalker et al. (12), which appeared as an important by-product of a nontrivial theory of “Pechucas gas of levels” developed in ref. 13. It gave rise to the relationship $\chi =(1-D_{2})/2$ (12), which linked the fractal dimension $D_{2}$ to the coefficient of proportionality, the spectral compressibility *χ*, between the level number variance and the mean number of levels in a spectral window containing critical states. This result appeared to be valid only for weak multifractality when $(1-D_{q})/q$ is the same for all *q*. A question, which remained open at that time, was which of the fractal dimensions $D_{q}$ would remain relevant for this type of relationship at an arbitrary strength of multifractality.

The answer to this question was conjectured by Bogomolny and Giraud (14), who demonstrated that a number of unconventional random-matrix models with long-range hopping obey the modified relationship by Chalker, Kravtsov, and Lerner (12), in which $(1-D_{2})/2$ is replaced by $1-D_{1}$. Shortly after, the conjecture was tested on a 2*d* generalization of a random-matrix model with long-range hopping (15). Later, it was shown (16, 17) that an important class of Toeplitz and Hankel random matrices exhibit critical level and eigenfunction statistics that approximately obey the same relation between the spectral compressibility *χ* and the fractal dimension $D_{1}$.

A key missing point of the above relations is to establish their relevance in physical models, for which the dominant processes are of short-range nature. Recently, advances in numerical approaches allowed for obtaining a fresh perspective on the critical properties of short-range disordered models, such as the Anderson models in two and three dimensions (18, 19). In particular, by comparing the scale-invariant critical dynamics at mid and late times, a recent study observed intriguing relations between the wavefunction and spectral properties at criticality (20), resembling the conjectured relations discussed above.

Here, we bridge the gap between exact relations in analytically tractable models and exact numerical results in finite physical models, and we establish the validity of a simple relation,

[1] $$\begin{array}{c} \chi +D_{1}=1, \end{array}$$

between the spectral compressibility *χ* and the wavefunction fractal dimension $D_{1}$. We note that in the quantum chaotic regime the relation is fulfilled trivially, since χ=0 and $D_{1}=1$. The same holds for the localized regime, where χ=1 and $D_{1}=0$. However, little is known about scenarios in which Eq. **1** holds nontrivially, i.e. in situations where both χ∈(0,1) and $D_{1}\in \left(0,1\right)$. The novelty of our work is the conjecture that such nontrivial validity of Eq. **1**, first introduced in ref. 14, is a universal property of a broad class of critical systems at the boundary between quantum chaos and localization. By universal property we have in mind the independence of the model’s dimensionality and symmetry class, and the locality of physical interactions; see also the discussion below.

We present a thorough numerical analysis to test Eq. **1** in Anderson models and the critical power-law random banded models (21). The latter model, though of long-range nature, was the simplest one in which the relation, Eq. **1**, was analytically demonstrated (14) in the limiting cases. It is also the model, in which the local spectrum is singular-continuous, with the local density of states being a random Cantor set (22). For the Anderson models, we focus on three, four, and five lattice dimensions with and without time-reversal symmetry. Remarkably, in all cases, we find that, to high precision, Eq. **1** is fulfilled. In addition, we confirm that Eq. **1** also holds for the critical orthogonal and unitary power-law random banded models, in the entire parameter range.

We then make a step forward and explore the validity of the available analytical predictions for *χ*. Surprisingly, we observe that *χ* in the power-law random banded model of unitary symmetry class can be well described by a simple surmise based on a modified Wigner-Dyson kernel, which exploits the analogy of level statistics with the position statistics of 1*d* fermions at a finite temperature (23–25). Exact numerical results establish the relevance of our expression. They show that the quantitative agreement is of similar accuracy as in the case of the well-known nearest level spacing (gap) distribution, where the Wigner surmise (1) describes the exact numerical distribution in ergodic systems to high accuracy. We apply the same approach to the averaged gap ratio *r* (26) and obtain good agreement with the exact numerical result.

Finally, we derive a relation between the fractal dimension $D_{1}$ and the average gap ratio *r*, which is valid for a broad class of critical systems. We check this relationship for the Anderson models in three, four, and five dimensions, as well as for the systems with semi-Poisson statistics (16, 17). In all the cases, we find good agreement of our theory with the corresponding numerical results.

## Models and Quantities Under Investigation

Below we provide information about the models under investigation. All of them exhibit a well-established single-particle eigenstate localization transition, enabling a detailed exploration of their critical properties. Further details can be found in *Materials and Methods*.

### Anderson Models.

We start by considering Anderson Models in d=3,4,5 spatial dimensions, corresponding to single-particle Hilbert space dimensions $N=L^{d}$, described by a Hamiltonian of the general form:

[2] $$\begin{array}{c} \hat{H}=-\sum_{\langle r_{j},r_{l}\rangle }t_{r_{j},r_{l}}({\hat{c}}_{r_{j}}^{†}{\hat{c}}_{r_{l}}+\text{h}.\text{c}.)+\sum_{r_{j}}h_{r_{j}}{\hat{n}}_{r_{j}}, \end{array}$$

where ${\hat{n}}_{r_{j}}\equiv {\hat{c}}_{r_{j}}^{†}{\hat{c}}_{r_{j}}$, $\langle r_{j},r_{l}\rangle$ denotes neighboring lattice sites, and $t_{r_{j},r_{l}}$ denotes the hopping element between those sites. We choose periodic boundary conditions. The onsite potentials $h_{r_{j}}$ are, for a given disorder strength *W*, drawn randomly from the uniform (“box”) probability distribution, $h_{r_{j}}\in \left[-W/2,W/2\right]$. This class of models is known to undergo a transition from single-particle quantum chaos to localization for d>2 (27–29).

In the simplest case of a real Hamiltonian and an isotropic lattice, $t_{r_{j},r_{l}}\equiv t$, with real parameter *t*, time-reversal symmetry is obeyed and the model’s statistics is, at weak disorder, expected to agree with predictions of the Gaussian orthogonal ensemble (GOE) for disorder strengths well below the transition. Thus we refer to these models as “orthogonal” Anderson models. The transition points are known (30) with high accuracy to lie at $W_{c}=16.54\;\left(3d\right),$$W_{c}=34.6\;\left(4d\right)$ and $W_{c}=57.3\;\left(5d\right)$.

To extend to systems of unitary universality class, we further study Anderson models of broken time-reversal symmetry to which we refer as “unitary” Anderson models, since their statistics is, at weak disoder, expected to agree with predictions of the Gaussian unitary ensemble (GUE). To break time-reversal symmetry, we follow two different approaches. In the first approach we consider random hopping phases (31, 32), i.e.,

[3] $$\begin{array}{c} t_{r_{j},r_{l}}\equiv t\;{\text{e}}^{i\varphi_{\mathrm{jl}}},\varphi_{\mathrm{jl}}\in \left[0,2\pi \right],\varphi_{\mathrm{lj}}=-\varphi_{\mathrm{jl}}, \end{array}$$

for the connected lattice sites $r_{j}$ and $r_{l}$. We study this version of the unitary Anderson model in up to five dimensions. While the transition for 3*d* and 4*d* is known from previous studies to emerge at $W_{c}=18.83$ and $W_{c}=37.5$, respectively (31, 32), we pinpoint the transition in five dimensions to $W_{c}=61.3$. For the second approach we dress the hopping term with a complex phase in d−2 dimensions to mimic the effect of a magnetic field, see *Materials and Methods* and Eq. **20** therein.

### Power-Law Random Banded Matrices.

Further we study power-law random banded (PLRB) matrices (21) given by the Hamiltonian

[4] $${\hat{H}}_{\text{PLRB}}=\sum_{i,j=1}^{N}h_{ij}{\hat{c}}_{i}^{†}{\hat{c}}_{j},$$

[5] $$h_{ij}=h_{ji}=\frac{\mu_{ij}}{{\left[1+{\left(\frac{N}{\pi }\sin(|i-j|\frac{\pi }{N})/b\right)}^{2a}\right]}^{1/2}},$$

where the matrix μ, with matrix elements $\mu_{\mathrm{ij}}$, is drawn either from the GOE, yielding the PLRB models of orthogonal universality class (GOE-PLRB), or from the GUE (GUE-PLRB). The sine term ensures periodic boundary conditions. This model exhibits a localization transition at the critical regime of a=1, which will be the focus of this study. The critical eigenstates exhibit strong multifractality for b≪1 and weak multifractality for b≫1 (10).

To ensure the criticality of states, we extract eigenvalues and eigenstates in all models from a narrow energy window in the middle of the spectrum, at $W=W_{c}$ in the Anderson models and at a=1 in the PLRB models. In all models, we denote the eigenvalue problem as $\hat{H}|E_{\alpha }\rangle =\varepsilon_{\alpha }|E_{\alpha }\rangle$. Following the unfolding procedure described in *Materials and Methods*, we set the mean level spacing *δ* to unity to obtain the unfolded eigenvalues $E_{\alpha }$.

### Level Number Variance and Spectral Compressibility.

The level number variance $\Sigma^{2}$ is a characteristic spectral property defined as

[6] $$\begin{array}{c} \Sigma^{2}\left(\Delta \right)={\langle n^{2}\rangle }_{H}-{\langle n\rangle \;}_{H}^{2}, \end{array}$$

where *n* denotes the number of levels (i.e. eigenvalues) lying in an energy interval of width Δ, measured in units of mean level spacing *δ*, while $\langle \cdots_{H}\rangle$ denotes the averaging over different Hamiltonian realizations. The spectral compressibility *χ* is commonly defined via the relation ${\text{Σ}}^{2}=\chi {\langle n\rangle }_{H}$. Correspondingly, we define an energy-dependent spectral compressibility χ(Δ) for random Hamiltonians as

[7] $$\begin{array}{c} \chi \left(\Delta \right)\;=\;{\langle \frac{{\langle n^{2}\rangle }_{H}-{\langle n\rangle \;}_{H}^{2}}{{\langle n\rangle }_{H}}\rangle }_{I}, \end{array}$$

where $\langle \cdots_{I}\rangle$ denotes the averaging over different interval positions around the middle of the spectrum, see *Materials and Methods* for further details on the numerical implementation.

**Fig. 1.**
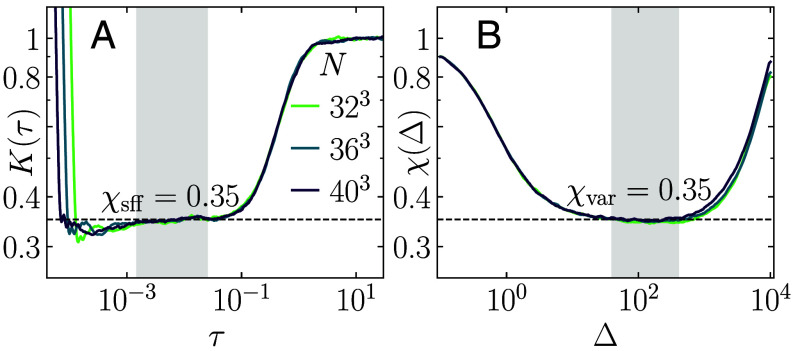
Comparison of the two numerical methods to extract the spectral compressibility. (*A*) SFF from Eq. **9**, and (*B*) the energy-depended spectral compressibility from Eq. **7** at the critical point $W_{c}=16.54$ of the 3*d* orthogonal Anderson model for three different system sizes *N*. The plateau values $\chi_{\text{sff}}$ and $\chi_{\text{var}}$ in panels (*A* and *B*), respectively, correspond to the spectral compressibility, extracted in the shaded intervals.

The peculiarity of the critical level statistics, first observed in refs. 5 and 7, is that in the limit when N→∞ is taken first and then Δ→∞, the spectral compressibility χ(Δ) tends to a constant value *χ*,

[8] $$\begin{array}{c} \chi =\underset{\text{Δ}\;\to \;\infty }{\lim}\left(\underset{N\;\to \;\infty }{\lim}\chi (\text{Δ})\right), \end{array}$$

where 0<χ<1. Away from criticality, it is known that χ=0 in the ergodic (1) and χ=1 in the localized regime. For a finite *N* in the actual numerical calculations, this implies a plateau in χ(Δ) for 1≪Δ≪N. The value of χ(Δ) at the plateau, for a sufficiently large *N*, then gives a good estimate of *χ*, see Fig. 1*B* for an example.

### Spectral form Factor and Spectral Compressibility.

Another commonly investigated spectral measure is given by the spectral form factor (SFF), defined as

[9] $$\begin{array}{c} K\left(\tau \right)=\frac{1}{Z}{\langle {\left|\sum_{\alpha =1}^{N}\eta \left(E_{\alpha }\right)\;{\text{e}}^{-2\pi iE_{\alpha }\tau }\right|}^{2}\rangle }_{H}, \end{array}$$

where time *τ* is taken in units of the inverse mean level spacing, *Z* normalizes the long-time value to one and *η* defines a Gaussian filtering function to reduce contributions from the spectral edges (33). The definition of the filtering function and further numerical details are elaborated in *Materials and Methods*.

It is known analytically (12) that in the thermodynamic limit, the spectral compressibility *χ* is connected to the SFF K(τ) via

[10] $$\begin{array}{c} \chi =\underset{\tau \;\to \;\infty }{\lim}\left(\underset{N\;\to \;\infty }{\lim}K(\tau )\right), \end{array}$$

see also *Materials and Methods* for details. In this work, we demonstrate that this relation allows to extract the spectral compressibility at the critical point also from finite-size numerical analyses. This is possible due to the emergence of a scale-invariant plateau in the SFF at intermediate times $\eta_{0}/N\ll \tau \ll 1$ ($\eta_{0}$ is the width of the filtering function) at criticality (18–20, 34, 35). Since the onset of this plateau drifts to τ→0 as N→∞, the plateau value allows to access the spectral compressibility very accurately even for finite system sizes. An illustrative comparison of the two methods to extract the spectral compressibility is shown in Fig. 1 at the critical point of the 3*d* orthogonal Anderson model.

### Fractal Dimensions.

We extract the fractal dimensions $D_{q}$ corresponding to the basis |{i}〉 spanned by site-local single-particle states $|i\rangle \equiv {\hat{c}}_{i}^{†}|\emptyset \rangle$, where |∅〉 denotes the vacuum state. To this end we first introduce the Shannon–von Neumann entropy (36) for a given eigenstate $|E_{\alpha }\rangle$,

[11] $$\begin{array}{c} S_{\text{SvN}}=-{\langle {\langle \sum_{i=1}^{N}|\langle i|E_{\alpha }\rangle {|}^{2}\ln{\left|\langle i|E_{\alpha }\rangle \right|}^{2}\rangle }_{\alpha }\rangle }_{H}, \end{array}$$

and the corresponding typical Rényi entropy (37),

[12] $$\begin{array}{c} S_{\text{R}}^{\left(q\right)}=\frac{1}{1-q}{\langle {\left\langle \ln P_{q,\alpha }^{-1}\right\rangle }_{\alpha }\rangle }_{H}, \end{array}$$

where in Eq. **12** the moments of the inverse participation ratios, $P_{q,\alpha }^{-1}$ for $|E_{\alpha }\rangle$, are defined as $P_{q,\alpha }^{-1}=\sum_{i=1}^{N}{\left|\langle i|E_{\alpha }\rangle \right|}^{2q}.$ The averaging over different Hamiltonian realizations, and different mid-spectrum eigenstates within a single realization, is denoted by ${\left\langle ...\right\rangle }_{H}$ and ${\left\langle ...\right\rangle }_{\alpha }$, respectively. We then extract the fractal dimension $D_{q}$ from a fit to the scaling ansatz,

[13] $$\begin{array}{c} S_{\text{R}}^{\left(q\right)}=D_{q}\ln N+c_{q}, \end{array}$$

in which for the special case q=1 the Rényi entropy should be replaced by the Shannon–von Neumann entropy. This work focuses on the fractal behavior $0<D_{q}<1$ associated with criticality (9), while, for all values of *q*, the ergodic regime is characterized by $D_{q}=1$ and the localized regime by $D_{q}=0$ (10).

## Testing the Conjecture

### Numerical Results.

**Fig. 2.**
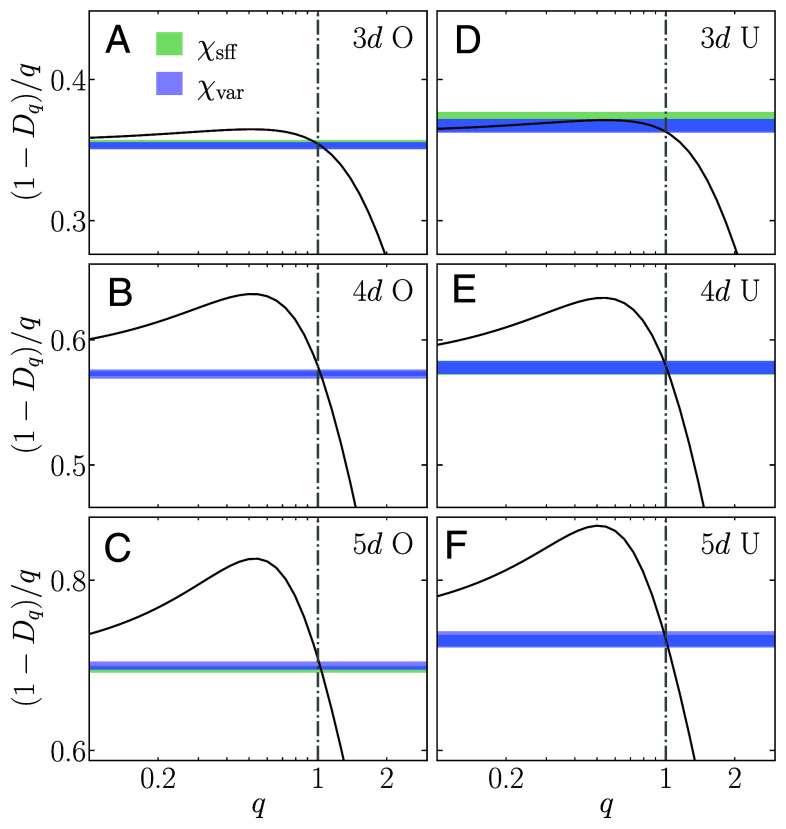
Relation between the scaled fractal dimension $(1-D_{q})/q$ (solid lines) and spectral compressibility *χ* (horizontal lines of finite width) versus *q* in the Anderson models of three, four, and five dimensions, belonging (*A*–*C*) to the orthogonal (O), and (*D*–*F*) to the unitary (U) universality class at the critical point. Here, the Anderson models of unitary universality class correspond to the definition given in Eq. **20** for three and four spatial dimensions (*D* and *E*), and to Eq. **3** for five dimensions (*F*). Spectral compressibilities are obtained for the largest available system size via the level number variance, Eq. **7**, and the SFF, Eq. **9**, labeled as $\chi_{\mathrm{var}}$ and $\chi_{\mathrm{sff}}$, respectively. The width of the horizontal lines denotes the estimate of their error bars.

We now test the connection between different fractal dimensions $D_{q}$ and the spectral compressibility *χ* in the various Anderson models, extracting *χ* for the largest available system size at the critical point using both the level number variance and the SFF. In Fig. 2, we plot the curves for $(1-D_{q})/q$ versus *q*, and find that the curves intersect with the horizontal line representing *χ* at the values of *q* that are, to high numerical precision, given by q=1. This provides strong evidence for the validity of the relation in Eq. **1** for all the Anderson models under consideration, both in the orthogonal and the unitary case, and for all considered spatial dimensions.

**Fig. 3.**
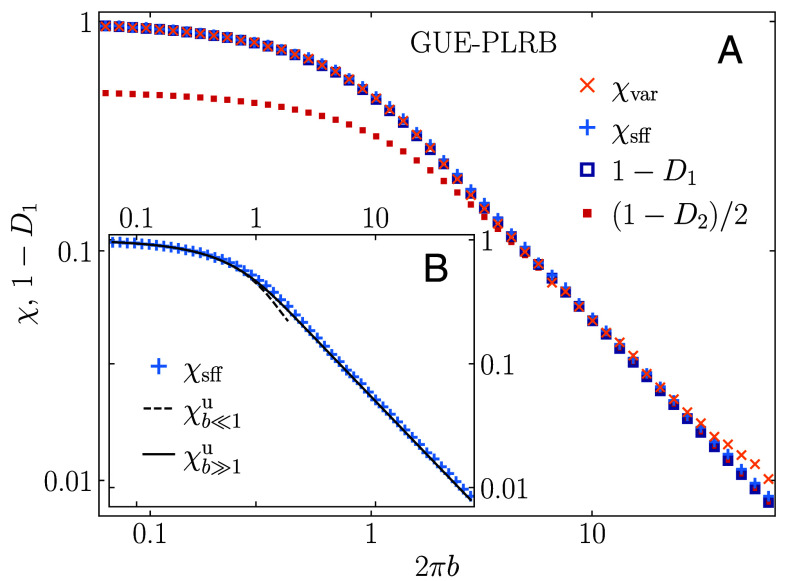
(*A*) Relation between the spectral compressibility *χ* (obtained via the level number variance, $\chi_{\mathrm{var}}$, and the SFF, $\chi_{\mathrm{sff}}$) and the subtracted fractal dimension $1-D_{1}$, at the critical manifold a=1 of the GUE-PLRB model, versus 2πb. We also plot $(1-D_{2})/2$ versus 2πb, matching the spectral compressibility only in the weak multifractality limit 2πb≫1. (*B*) Comparison of the numerically obtained $\chi_{\mathrm{sff}}$ and the theoretical predictions $\chi_{b\ll 1}^{u}$ and $\chi_{b\gg 1}^{u}$, given by Eqs. **14** and **16**, respectively.

We then extend our analysis to the PLRB models of orthogonal and unitary symmetry class in the broad regime of the tuning parameter *b*. Results in Fig. 3*A* for the GUE-PLRB model, and in *Materials and Methods* for the GOE-PLRB model, give rise to the accurate agreement between $1-D_{1}$ and *χ* for all *b*. We also note that the more general relation $\chi =(1-D_{q})/q$ holds true in the weak multifractality limit 2πb≫1, as exemplarily illustrated in Fig. 3*A* for q=2 that corresponds to the original relation from ref. 12.

Our thorough numerical analysis hence strongly supports validity of the relation in Eq. **1** in both local and nonlocal models at criticality, irrespective of the symmetry class and lattice dimensionality.

### Limiting Cases: Analytical Insights.

We corroborate our numerical results with analytical insights in certain limiting cases. In particular, analytical tools are available for the PLRB models to obtain exact solutions for the spectral compressibility in the limits 2πb≪1 and 2πb≫1, corresponding to the limits of strong and weak multifractality, respectively. In the limit of strong multifractality, 2πb≪1, the PLRB matrices are almost diagonal and the spectral compressibility was calculated as a perturbative expansion in the number of resonating energy levels up to order $b^{2}$ for the GUE-PLRB and up to linear order in *b* for the GOE-PLRB model (38, 39).^*^ The results of this expansion are

[14] $$\begin{array}{l} \chi^{\text{u}}(b)=\chi_{b\ll 1}^{\text{u}}(b)+O(b^{3})\\ \chi_{b\ll 1}^{\text{u}}(b)=1-\sqrt{2}(\pi b)+\frac{4}{\sqrt{3}}(2-\sqrt{3}){\left(\pi b\right)}^{2}, \end{array}$$

[15] $$\begin{array}{l} \chi^{\text{o}}(b)=\chi_{b\ll 1}^{\text{o}}(b)+O(b^{2})\\ \chi_{b\ll 1}^{\text{o}}(b)=1-\sqrt{2}(2b), \end{array}$$

where $\chi^{\text{u}}$ and $\chi^{\text{o}}$ correspond to the spectral compressibilities in the GUE-PLRB and GOE-PLRB models, respectively.

In the limit of weak multifractality, 2πb≫1, the PLRB models can be mapped onto the nonlinear sigma model (21). Then one can apply the relationship between the classical and quantum spectral determinants (40) and compute (24, 25) the density-of-states correlation function $\tilde{K}\left(\omega \right)$, which can be thought of as the Fourier transform of the SFF K(τ), see *Materials and Methods*. Integrating $\tilde{K}\left(\omega \right)$ over *ω*, one obtains, according to Eq. **10**, the spectral compressibility *χ* for the unitary and the orthogonal ensembles, respectively,

[16] $$\begin{array}{cc} \chi_{b\gg 1}^{\text{u}}\left(b\right) & =\frac{1}{4\pi b}+1-\coth\left(4\pi b\right), \end{array}$$

[17] $$\begin{array}{cc} \chi_{b\gg 1}^{\text{o}}\left(b\right) & =\frac{1}{2\pi b}+1-\coth\left(2\pi b\right). \end{array}$$

Importantly, Eqs. **16** and **17** can also be obtained from the Wigner-Dyson formalism (1) with the modified kernel,

[18] $$\begin{array}{c} F\left(x-y\right)=\frac{\sin(\pi (x-y))}{4b\;\sinh(\pi (x-y)/4b)}, \end{array}$$

see *Materials and Methods* for further details. This kernel corresponds to the position statistics of 1*d* free fermions at a finite temperature T=1/4b. In particular, the density correlation function is given by $1-F{\left(x-y\right)}^{2}$, like the eigenvalue density correlation function in the GUE-PLRB model. This establishes a correspondence between the critical level statistics and the position statistics of 1*d* fermions at a finite temperature. At weak multifractality (corresponding to 1*d* free fermions at small temperature), this analogy holds for all three Dyson universality classes (25).

In passing, we note that also the fractal dimensions $D_{q}$ in the critical PLRB models were obtained analytically up to the first order in *b* and $b^{-1}$ for the limiting cases of strong and weak multifractality, respectively (14, 38). In both limits the relation $\chi +D_{1}=1$ was thereby analytically confirmed up to first order. However, no analytical predictions are available for $D_{1}$ in the intermediate regime, which is the most relevant for physical systems.

### Toward a General Relationship.

**Fig. 4.**
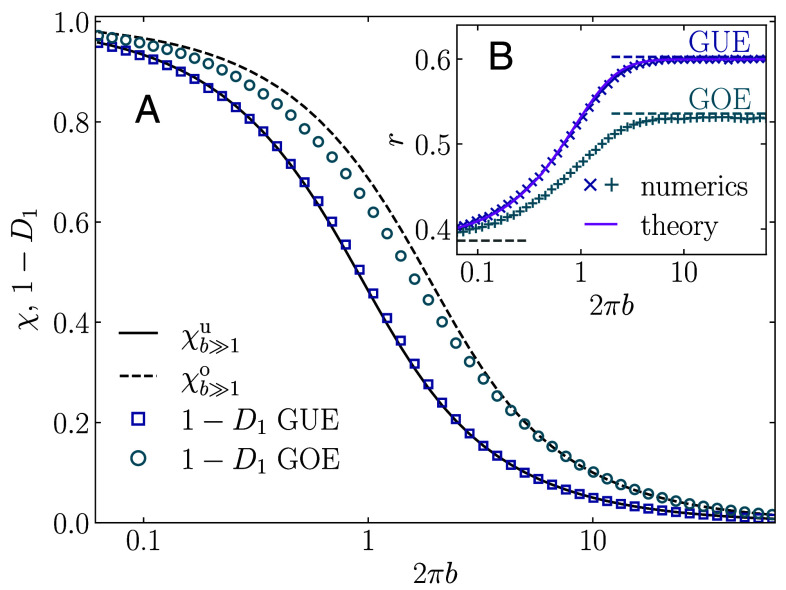
(*A*) Spectral compressibility *χ* and the subtracted fractal dimension $1-D_{1}$ versus 2πb, at the critical manifold a=1 of the PLRB model in the unitary (GUE) and the orthogonal universality (GOE) class. Numerical results for $1-D_{1}$ are displayed by symbols and the analytical results for $\chi_{b\gg 1}^{u}$ and $\chi_{b\gg 1}^{o}$, see Eqs. **16** and **17**, respectively, are displayed as lines. (*B*) Average gap ratio *r* in the PLRB models versus 2πb, at a=1. Symbols are numerical results and the line for the GUE-PLRB model denotes *r* obtained from the Wigner-Dyson formalism of ref. 41 with the modified kernel F(ω) from Eq. **18**.

Surprisingly, we find that further steps can be made connecting the closed-form expressions for the limiting cases to the nonperturbative regime. Specifically, we observe in the GUE-PLRB model an almost perfect agreement between the analytical prediction for $\chi_{b\gg 1}^{u}$ from Eq. **16** and the numerical results for the spectral compressibility *χ* and hence with the subtracted fractal dimension, $1-D_{1}$, in a broad regime of the parameter *b*. These results are shown on a log–log scale in Fig. 3*B* and on a linear-log scale in Fig. 4*A*.

The accuracy of our surmise is remarkable provided that by derivation, Eq. **16** is only valid for b≫1 where the nonlinear sigma-model applies, see the discussion in ref. 25. Moreover, Eq. **16** does not have exactly the same expansion in powers of *b* as the exact expansion in the opposite limit b≪1, given by Eq. **14**. This means that the accuracy of our surmise in Eq. **16** is similar to the accuracy of the celebrated Wigner surmise $P_{\text{W}}\left(s\right)$ for the distribution P(s) of closest gaps *s*, i.e., $P_{\text{W}}\left(s\right)=32{\left(s/\pi \right)}^{2}\exp\left[-4s^{2}/\pi \right]$. While $P_{\text{W}}\left(s\right)$ is very close to the exact gap distribution given by the corresponding Fredholm determinant (1), it has a different coefficient in front of $s^{2}$ at small *s* and a different coefficient in the Gaussian behavior at large *s*.

It is also known that the corresponding Wigner surmise for the GOE has more significant deviations from the exact P(s) than that for the GUE. The same is true for $\chi_{b\gg 1}^{o}$ from Eq. **17**, which is compared to the exact numerical results for the GOE-PLRB model in Fig. 4*A*. While the agreement between the latter and $\chi_{b\gg 1}^{o}$ is perfect at large 2πb, the deviations become significant at intermediate values of 2πb.

To further corroborate that the analogy with free fermions at a finite temperature gives a very accurate surmise for different spectral statistics in the GUE case, we compute the average gap ratio *r* by the Fredholm determinant formalism (1, 41) based on the same modified kernel, Eq. **18**, as the one that was employed to compute Eq. **16**. Again we find an almost perfect agreement with the numerically obtained *r* (see Eq. **22** for the definition of *r*) across the whole critical manifold, see Fig. 4*B*. The details of this semianalytical calculation will be published elsewhere. We hence conclude that the Wigner-Dyson theory based on a modified kernel, Eq. **18**, gives a very accurate prediction for different spectral statistics of the GUE-PLRB model across the entire critical manifold.

**Fig. 5.**
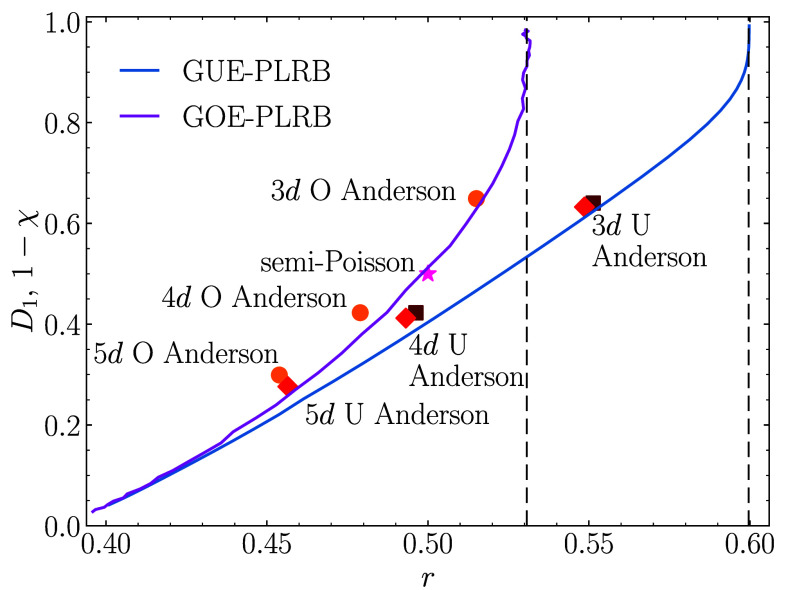
Universal functions $D_{1}\left(r\right)$ for the critical models of the unitary (blue curve) and orthogonal (magenta curve) symmetry class. They are obtained semianalytically (as 1-*χ*) for the GUE-PLRB model as explained in the text and numerically for the GOE-PLRB model. Symbols: numerical results for different critical models (points with the statistical error bars corresponding to their sizes): 3*d*, 4*d*, and 5*d* Anderson models of orthogonal (dots) and unitary symmetry class (diamonds for the model in Eq. **3**, squares for the model in Eq. **20**). The star corresponds to systems with the “semi-Poisson” distribution of gaps where $D_{1}=r=1/2$ (16, 17). The vertical dashed lines are the Wigner-Dyson limits r=0.5997 and r=0.5307.

Finally, we derive a relationship between the fractal dimension $D_{1}$ and the average gap ratio *r*, which allows us to demonstrate its universality for different critical models, both local and nonlocal, with and without time-reversal symmetry. The derivation is carried out semianalytically for the GUE-PLRB model, combining Eq. **16** with the results for *r* obtained by applying the Wigner-Dyson formalism with the modified kernel, Eq. **18**. Indeed, the functions χ(b) and r(b) parametrically define the function χ(r). Then, using the relation $1-D_{1}=\chi$ from Eq. **1**, we can express $D_{1}$ in terms of *r*. The same operation for the GOE-PLRB model is done using numerical results for χ(b) and r(b). The results are shown in Fig. 5.

We test the universality of the functions $D_{1}\left(r\right)$ by calculating the values of $D_{1}$ and *r* at the critical points of the Anderson models in 3*d*, 4*d*, and 5*d*, both in the orthogonal and unitary symmetry class, as well as by adding the point $D_{1}=r=1/2$ that characterizes systems with semi-Poisson statistics, see, e.g., refs. 16 and 17. The results (symbols) in Fig. 5 show a reasonably good agreement with our predictions (solid lines). Nevertheless, the agreement becomes less perfect in higher-dimensional Anderson models. We attribute these deviations to systematic errors that emerge in numerical calculations for finite systems, in which the accessible linear dimensions $L=\sqrt[{d}]{N}$ decrease with the increase of dimensionality. We note that the dependence of $D_{1}\left(L\right)$ near criticality typically exhibits a shallow minimum (42–45). Hence, one may observe a decrease of $D_{1}\left(L\right)$ at the accessible sizes *L*, while at even larger sizes, $D_{1}\left(L\right)$ may start to grow toward the ergodic limit $D_{1}=1$. Therefore the numerical results at limited system sizes may suggest the system’s tendency toward localization, while it is actually still in the delocalized phase. Consequently, underestimating the critical value $W_{c}$ gives rise to a systematic overestimate of $D_{1}\left(W_{c}\right)$, which is consistent with the observations in Fig. 5.

Using the analytically derived universal function $D_{1}\left(r\right)$ one may also predict the values of *r* for another long-disputed transition, namely, the integer quantum Hall effect transition at the center of the Landau band. Carrying out a high-precision computation on the Chalker-Coddington network (46), the value of $D_{1}$ for this transition was obtained to be $D_{1}=0.8702$, and a field-theoretical calculation by Zirnbauer (47) predicted the critical value $D_{1}=7/8$. The (unitary) universal function derived here gives r=0.5962 for $D_{1}=0.8702$ and r=0.5966 for $D_{1}=7/8$. In future, it would be interesting to compare this prediction with the numerical results on the Chalker-Coddington networks.

### Eigenfunction Support Set and the Special Role of *D*_1_.

A priori it is not clear why, out of all fractal dimensions, $D_{1}$ is the one which connects to the spectral compressibility via the relation $\chi +D_{1}=1$, even for strong multifractality. While it is out of scope for this work to deliver a stringent argument or mathematical proof for this relation, we want to note that there are possible arguments why $D_{1}$ is special from other fractal dimensions. A special role of fractal dimension $D_{1}$ in Eq. **1** is related with the fact that $D_{1}$ determines the eigenfunction support set volume. The definition of the support set volume $M_{\epsilon }$ is

[19] $$\begin{array}{c} \sum_{i=1}^{M_{\epsilon }}|\left\langle i|E_{\alpha }\right\rangle {|}^{2}\leq 1-\epsilon \leq \sum_{i=1}^{M_{\epsilon }+1}{\left|\left\langle i|E_{\alpha }\right\rangle \right|}^{2}. \end{array}$$

In other words, the support set is the minimal set of sites that yield a nonvanishing contribution to the norm, i.e., ϵ<1. In ref. 48 it was argued that $M_{\epsilon }=N^{D_{1}}$ in the limit N→∞. We note however that this convergence can be slow, hence the support set volume does not necessarily pose a suitable method to obtain the fractal dimension in finite-size numerics. Yet, in view of the crucial role played by normalization and completeness conditions of quantum mechanics, the fractal dimension $D_{1}$ is clearly special.

## Discussion

Our work establishes the generality of the relationship $\chi +D_{1}=1$ between the spectral compressibility and the wavefunction fractal dimension, for physical models in various dimensions, across different symmetry classes. Moreover, we also find indications of relations between the spectral and wavefunction properties beyond Eq. **1**, triggered by the relevance of the Wigner-Dyson formalism with the modified kernel in a broad regime of the critical manifold. This opens doors for further exploration of spectral properties beyond compressibility, such as the average gap ratio.

Our results also raise several outstanding questions for future research. One of them is the strict definition, and possibly the uniqueness, of the preferred basis in which the wavefunction fractal dimension takes a minimal value. By definition, the preferred basis should be independent of the Hamiltonian realization and thus the energy basis is excluded.

The preferred basis of the models studied here appears to coincide with the “natural” basis in which the model is formulated. This is certainly not always the case, and a counterexample may be given by the ensemble of Toeplitz matrices from refs. 16 and 17. These matrices are formulated in the basis in which their entries are $H_{\mathrm{nm}}=f(|n-m|)$, with f(n) being i.i.d. Gaussian random variables. However, the preferred basis for these models is the momentum basis, in which eigenfunctions show multifractal statistics.

Further, one can also imagine a continuous family of preferred bases with the same minimal $D_{1}$, such as a “mexican hat” of preferred bases. In this case, the averaging over such bases does not eliminate the deviation of spectral statistics from the Wigner-Dyson statistics, as demonstrated in ref. 23.

All critical systems studied here contain parameters of the Hamiltonian that are independent of the system size, and the local spectrum in the preferred basis is a random Cantor set (22). These appear to be sufficient conditions for Eq. **1** to hold. In such systems the local density-of-states correlation function is a power law, with an exponent that belongs to the interval (0,1) for all energies down to the level spacing (22), thus connecting the large energy scale properties (spectral compressibility) and the small energy scale properties, such as the average gap ratio.

Another outstanding question is the generalization of our results to interacting systems, and their possible relevance for quantum dynamics. From the perspective of spectral properties in interacting systems, recent work has suggested that the dynamics of the spectral form factor may provide information about the many-body spectral compressibility (20, 34). Information about the fractal dimension $D_{2}$ of the many-body wavefunctions can also be extracted from the power law decay of survival probability of many-body wavefunctions (35).

However, for many-body systems, a “natural” basis is not obvious and the problem of a preferred basis is highly nontrivial. A recent study (49) demonstrated that the fractal dimensions $D_{1}$ and $D_{2}$ are different for a spin-1/2 XXZ chain in the configurational (bitstring) basis, and in the Fock basis built by Anderson orbitals, taking smaller values in the latter. This means that the Fock basis is closer to the preferred one than the configurational basis. Some recent attempts to approach the problem of preferred basis in many-body systems are described in refs. 50 and 51.

Establishing possible relationships between spectral and wavefunction features in interacting quantum systems may provide new impetus to the analysis of critical properties at the many-body ergodicity breaking transitions, including the many-body localization transition that has recently experienced a lively discussion about the position of the transition point in certain paradigmatic models (52).

## Materials and Methods

### Anderson Models in Unitary Universality Class.

We consider two different instances of Anderson models belonging to the unitary universality class. The first, corresponding to random hopping phases, was defined in Eq. **3** in the main text. For the second option, we dress the hopping term with a complex phase in d−2 dimensions according to

[20] $$\begin{array}{cccc} t_{r,r+x} & \equiv t, & & t_{r,r+z}\equiv t{\text{e}}^{2\pi i\phi x},\\ t_{r,r+y} & \equiv t, & & t_{r,r+w}\equiv t{\text{e}}^{2\pi i\phi (x+y)}, \end{array}$$

where the fourth spatial direction w is only present in the case of four dimensions. In the 3*d* case, this definition corresponds to the presence of a magnetic field of flux *ϕ* in y direction. We choose ϕ=1/4 throughout this study. For this strength of the magnetic field a transition point of $W_{c}=18.38$ was found in a previous study (53). For the 4*d* case, no previous studies of the transition are available, although the coupling of fluxes as in Eq. **20** was discussed in the context of topological insulators (54). Therefore, we carry out a scaling analysis to extract the transition point, yielding $W_{c}=37.1$, see the text below for further details. Due to the limited available system sizes in higher dimensions, we use both periodic and antiperiodic boundary conditions in the 4d case, while we refrain from a further generalization of this model to 5*d*, since the accessible system sizes for which periodic or antiperiodic boundary conditions can be chosen are limited.

### Results for GOE-PLRB Model.

**Fig. 6.**
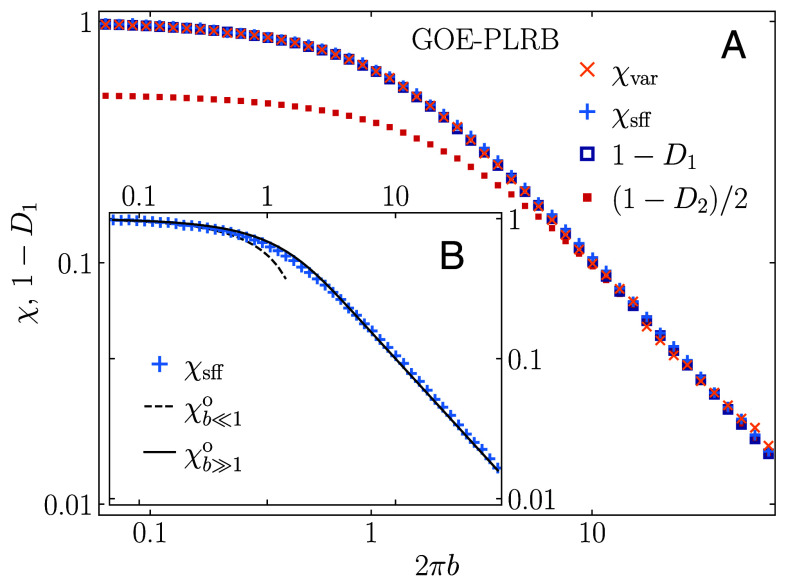
(*A*) Relation between the spectral compressibility *χ* (obtained via the level number variance, $\chi_{\mathrm{var}}$, and the SFF, $\chi_{\mathrm{sff}}$) and the subtracted fractal dimension $1-D_{1}$, at the critical manifold of the GOE-PLRB model, versus 2πb. We also plot $(1-D_{2})/2$ versus 2πb, matching the spectral compressibility only in the weak multifractality limit 2πb≫1. (*B*) Comparison of the numerically obtained $\chi_{\text{sff}}$ and the theoretical predictions $\chi_{b\ll 1}^{o}$ and $\chi_{b\gg 1}^{o}$, given by Eqs. **15** and **17**, respectively.

We repeat the same analysis, as shown in Fig. 3, for the PLRB model of orthogonal universality class. The results are displayed in Fig. 6. Again we observe validity of the relation $\chi +D_{1}=1$ across the whole critical manifold.

### Determination of Transition Points.

**Fig. 7.**
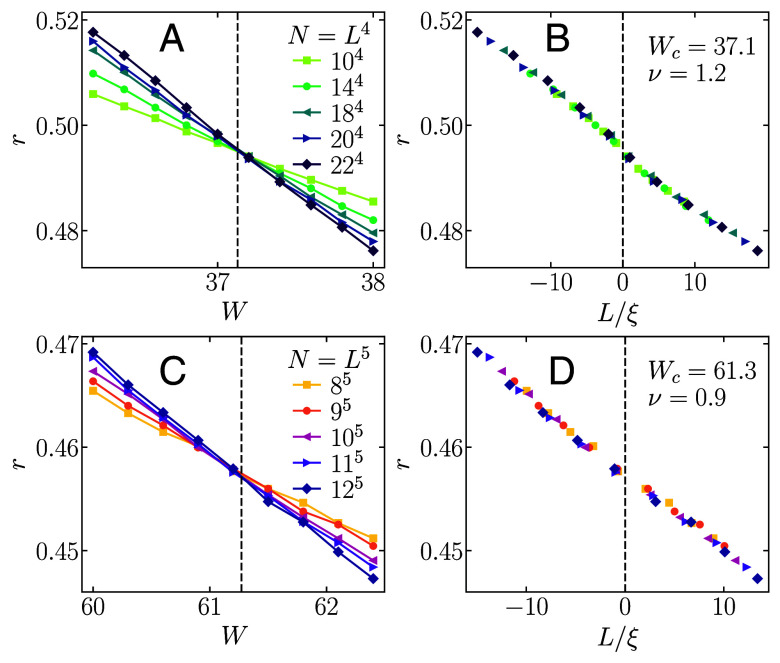
Determination of the transition points for the unitary Anderson models using the average gap ratio *r*, see Eq. **22**, as the transition indicator. (*A* and *B*) 4*d* unitary Anderson Model from Eq. **20**. (*C* and *D*) 5*d* unitary Anderson Model from Eq. **3**. Panels (*A* and *C*) show the results for *r* versus *W* for different total system sizes $N=L^{d}$. In panels (*B* and *D*) we show the optimal scaling collapse of *r* versus L/ξ, where *L* denotes the linear size and $\xi =\mathrm{sign}\left(W-W_{c}\right){\left|W-W_{c}\right|}^{-\nu }$. The optimal scaling collapse is obtained using the cost function minimization approach described in refs. 33 and 55. Vertical dashed lines in all panels correspond to the transition point $W=W_{c}$, and the extracted values of $W_{c}$ and *ν* are given in panels (*B* and *D*).

While the transition points of the Anderson models in the orthogonal symmetry class, as well as in certain lattice geometries in the unitary symmetry class, are known to high accuracy (30), we are not aware of any comparable analyses of the 4*d* unitary Anderson model described by Eq. **20**, and the 5*d* unitary Anderson model with random hopping phases from Eq. **3**. Therefore, we carry out a scaling analysis to extract the transition point in these models by applying the cost function minimization approach using the average gap ratio *r*, defined in Eq. **22**, as the transition indicator. Details of the method can be found in refs. 33 and 55. The results for *r* and the corresponding optimal scaling solutions are shown in Fig. 7. We find the transition point at $W_{c}\approx 37.1$ for the 4*d* unitary Anderson model from Eq. **20** and at $W_{c}\approx 61.3$ for the 5*d* unitary Anderson model from Eq. **3**.

### Details of Numerical Calculations.

Here we provide details about the numerical algorithms we used, the averaging over eigenstates and Hamiltonian realizations, the spectral unfolding, and the two different methods to extract the spectral compressibility, i.e., the level number variance and the spectral form factor.

#### Numerical methods.

The calculation of level number variance and spectral form factor requires access to a great fraction, respectively all of the eigenvalues for the model under investigation. Therefore, we obtain them using exact diagonalization routines. To calculate the average gap ratio, as well as the fractal dimensions, it suffices to determine a finite number of eigenstates in the middle of the spectrum. To this end we use the method of Polynomial-filtered Exact Diagonalization (56), allowing us to reach large system sizes for the Anderson Models. Since this method requires sparsity of the Hamiltonian matrix, it is not applicable to the dense PLRB models, for which we resort to exact diagonalization instead.

#### Averaging over eigenstates and Hamiltonian realizations.

In general, for a single Hamiltonian realization, the averages are carried out over the middle of the spectrum. The Anderson models exhibit a mobility edge for $W<W_{c}$, i.e., there exists an energy $E_{c}$ that separates localized and extended states. With increasing disorder strength *W*, the mobility edge moves quickly from the edges of the spectrum to its center, so that almost all the states are either extended or localized, the transition being extremely sharp at $W\approx W_{c}$. At the transition point $W_{c}$ and close to the mobility edge, there is a divergent critical length $\xi \propto |W-W_{c}{|}^{-\nu }$ (in the center of the energy band) or $\xi \propto |E-E_{c}{|}^{-\nu }$ (close to the mobility edge $E_{c}$), such that for L<ξ all the eigenstates are critical and exhibit multifractality.

The maximal detuning from the critical disorder strength $W_{c}$, or from the mobility edge $E_{c}$, to observe the critical states is $|1-W/W_{c}|∼|1-E/E_{c}|∼L^{-\frac{1}{\nu }}$. In units of the mean level spacing, δ∝1/N, it is therefore proportional to $L^{d-\frac{1}{\nu }}≳L^{\frac{1}{\nu }}$, which, according to the Harris criterion dν>2 (57), is a macroscopically large number in the large-*L* limit. So, despite the spectral window of the critical states shrinking to zero in the large-*L* limit, the number of levels in this window goes to infinity, allowing to define the critical spectral and eigenfunction statistics.

The actual number of Hamiltonian realizations we average over and the system sizes for all the investigated models can be seen in Table 1. We use the same system sizes and number of Hamiltonian realizations for the case of orthogonal and unitary universality class.

**Table 1.** Details of numerical simulations

Model	Quantity	System sizes *N*	$n_{R}$ ^*^	$n_{S}$ ^†^
3*d* And	*χ* $r,D_{1}$	32^3^, 36^3^, 40^3^ 24^3^, 32^3^, 40^3^, 48^3^, 56^3^, 64^3^	1,000 2,000	500
4*d* And	*χ* $r,D_{1}$	12^4^, 14^4^, 16^4^ 12^4^, 14^4^, 16^4^, 18^4^, 20^4^, 22^4^	2,000 5,000	500
5*d* And	*χ* $r,D_{1}$	7^5^, 8^5^, 9^5^ 8^5^, 9^5^, 10^5^, 11^5^, 12^5^, 13^5^	2,000 5,000	500
PLRB	*χ*	2k, 4k, 6k, 8k, 10k, 12k, 14k, 16k, 18k, 20k	1,000 500	
	$r,D_{1}$	2k, 4k, 6k, 8k, 10k, 12k, 14k, 16k, 18k, 20k	1,000 500	10% of *N*

^*^Number of different Hamiltonian realizations.

^†^Number of considered eigenstates per Hamiltonian realization.

#### Unfolding.

Prior to calculating the spectral form factor or the level number variance, we set the local mean level spacing to unity at all energy densities by unfolding the spectrum. To this end, starting from the exact eigenvalues $\varepsilon_{\alpha }$ for a given Hamiltonian realization, we construct the step function $G\left(\varepsilon \right)=\sum_{\alpha =1}^{N}\Theta \left(\varepsilon -\varepsilon_{\alpha }\right)$ and fit it with a polynomial of low order *n* (n=3 for Anderson models and n=5 for the PLRB models). The resulting fitting function $g_{n}\left(\varepsilon \right)$ yields the unfolded eigenvalues as $E_{\alpha }=g_{n}\left(\varepsilon_{\alpha }\right)$.

#### Details of spectral form factor calculations.

To reduce contributions from the spectral edges, we introduce a Gaussian filtering factor η(E) in the definition of the spectral form factor, Eq. **9**. For a given Hamiltonian realization yielding the unfolded eigenvalues $E_{\alpha }$, the filtering function is defined as

[21] $$\begin{array}{c} \eta \left(E\right)\;=\;\exp\left(-\frac{{\left(E_{\alpha }-\overset{¯}{E}\right)}^{2}}{2\;\eta_{0}\Gamma^{2}}\right), \end{array}$$

where $\overset{¯}{E}$ and $\Gamma^{2}$ denote the mean energy, respectively the energy variance, for the given Hamiltonian realization. The factor $\eta_{0}$ affects the width of the Gaussian and thereby the effective filtering strength. A priori, there is no best choice for $\eta_{0}$ since this would require perfect knowledge about the mobility edge and boundary effects for a given finite system. Empirically we find that for the Anderson models a filter using $\eta_{0}\in \left[0.3,0.7\right]$ yields the broadest plateau at the transition point, while for the PLRB models a filter using $\eta_{0}=0.3$ poses a suitable choice. The error estimate provided in Fig. 2 for the plateau value of the spectral form factor, and hence for the spectral compressibility, is based on a scan through the different filtering function widths and slightly different conceivable placements of the time-interval to extract the plateau value.

#### Details of level number variance calculations.

In our numerical calculations of the energy-dependent spectral compressibility via the level variance, as defined in Eq. **7**, we proceed in three steps. Starting from the unfolded spectrum for a given Hamiltonian realization, we first cut away a fraction of eigenvalues at the boundaries. We find that for the Anderson models, larger parts of the spectrum have to be considered to optimally unveil the plateau, corresponding to a cutoff of 10% to 25% of states at both ends of the spectrum, whereas for the PLRB models, a more narrow focus on only 20% of states in the middle of the spectrum works best.

In the next step, after the cutoff of spectral edges, we draw one box of width Δ in units of mean level spacing *δ* (∼1 after unfolding) for each Hamiltonian realization and place it within the remaining middle part of the spectrum. Empirically we find that it is beneficial to vary the midpoints from realization to realization by random displacements. In the Anderson models, in contrast to the PLRB models, a higher degree of randomness for the placement of midpoints is necessary to unveil the plateau optimally. Having obtained the number of levels within the box for all realizations, we calculate their variance and divide by the mean number of levels. Here, we want to note that, even for the unfolded spectrum it proves beneficial to divide by the mean number of levels across all realizations instead of the interval width. We argue that this is caused by the density of states, which is not perfectly flat even after the unfolding procedure.

In the last step, we repeat the procedure 20 times for differently chosen interval positions in each realization around the middle of the spectrum and average over the resulting spectral compressibility, to reduce fluctuations.

The error estimate provided in Fig. 2 for the spectral compressibility, obtained from the level number variance, is based on a scan through different sizes of the initial boundary cutoff, the amount of randomness in the placement of interval midpoints, and slightly different conceivable intervals to extract the plateau value.

### Gap Ratio.

A useful measure to characterize the localization transitions is the average gap ratio *r*, defined as (26)

[22] $$\begin{array}{c} \begin{array}{cc} r & ={\langle \overset{¯}{r}\rangle }_{H},\overset{¯}{r}=\langle {r_{\alpha }\rangle }_{\alpha },\\ r_{\alpha } & =\frac{\min(\varepsilon_{\alpha +1}-\varepsilon_{\alpha },\varepsilon_{\alpha }-\varepsilon_{\alpha -1})}{\max(\varepsilon_{\alpha +1}-\varepsilon_{\alpha },\varepsilon_{\alpha }-\varepsilon_{\alpha -1})}, \end{array} \end{array}$$

where $\langle \cdots_{H}\rangle$ and $\langle \cdots_{\alpha }\rangle$ denote averaging over different Hamiltonian realizations and the states around the middle of the spectrum, respectively, and the $\varepsilon_{\alpha }$ denote the exact Hamiltonian eigenvalues. We note, that calculating the gap ratio does not require the prior unfolding of the spectrum since the gap ratio is insensitive to the underlying density of states.

For the Wigner-Dyson GOE and GUE random-matrix ensembles, the distribution of $r_{\alpha }$ and its average value *r* can be exactly computed for any *N* by the Fredholm determinant formalism described in refs. 1 and 41. In ref. 41 a simple and rather precise surmise was derived using a simplified approach considering only a 3×3 matrix Hamiltonian. Unfortunately, this simplified approach is suitable only for the Wigner-Dyson kernel, Eq. **18**, at b→∞, as it is intimately related with the incompressibility of the system’s levels, χ=0.

In order to analytically compute the average gap ratio *r* in the limit N→∞ for an arbitrary *b* in the kernel from Eq. **18**, we employed the exact Fredholm determinant formalism as described in refs. 1 and 41. The result of the calculation of the function r(b) for the GUE-PLRB model is presented in Fig. 4*B*. Remarkably, it matches perfectly with the corresponding function found by exact numerics.

### Relation Between the Global Density of States Correlation Function and the Spectral Form Factor.

Consider the spectral form factor as defined in Eq. **9**:

[23] $$\begin{array}{cc} & K\left(\tau \right)=\frac{1}{Z}{\langle \sum_{\alpha ,\beta =1}^{N}\eta \left(E_{\alpha }\right)\eta \left(E_{\beta }\right){\text{e}}^{-2\pi i(E_{\alpha }-E_{\beta })\tau }\rangle }_{H}\\ & =\frac{1}{Z}\int d\omega e^{2\pi i\omega \tau }\int dE\;\eta \left(E\right)\eta \left(E-\omega \right)\;{\langle \rho (E)\rho (E-\omega )\rangle }_{H}, \end{array}$$

where all energies are measured in units of the mean level spacing and

[24] $$\begin{array}{c} \rho \left(E\right)=\sum_{\alpha =1}^{N}\delta \left(E-E_{\alpha }\right) \end{array}$$

is the (global) density of states (DoS). One can see that K(τ) is the Fourier transform of

[25] $$\begin{array}{c} \frac{1}{Z}\int dE\;\eta \left(E\right)\eta \left(E-\omega \right)\;G\left(\omega ,E\right), \end{array}$$

where G(ω,E) is the correlation function of the (global) DoS:

[26] $$\begin{array}{c} G\left(\omega ,E\right)={\langle \sum_{\alpha =1}^{N}\delta \left(E-E_{\alpha }\right)\sum_{\beta =1}^{N}\delta \left(E-\omega -E_{\beta }\right)\rangle }_{H}. \end{array}$$

Now consider the function G(ω,E) and its relation with the level number variance $\Sigma^{2}$ in an energy window Δ centered at E=0. The latter is given by definition

[27] $$\begin{array}{c} \Sigma^{2}={\langle \{\int_{-\Delta /2}^{\Delta /2}dE\left[\rho \left(E\right)-{\langle \rho (E)\rangle }_{H}\right]{\}}^{2}\rangle }_{H}. \end{array}$$

Using the definition of the DoS we obtain:

[28] $$\begin{array}{c} \Sigma^{2}={\langle \{\int_{-\Delta /2}^{\Delta /2}dE\sum_{\alpha =1}^{N}\left[\delta \left(E-E_{\alpha }\right)-{\langle \delta (E-E_{\alpha })\rangle }_{H}\right]{\}}^{2}\rangle }_{H} \end{array}$$

and rewrite it as

[29] $$\begin{array}{c} \Sigma^{2}=\int_{-\Delta /2}^{\Delta /2}dE\int_{-\Delta /2}^{\Delta /2}dE^{\prime }\;\left[G\left(E-E^{\prime },E\right)-{\left\langle \rho \left(E\right)\right\rangle }_{H}{\left\langle \rho \left(E^{\prime }\right)\right\rangle }_{H}\right]. \end{array}$$

At small (compared to the total spectral band-width) width of the filtering function η(E) and corresponding small Δ and *ω* the correlation function G(ω,E) is approximately independent of *E*:

[30] $$\begin{array}{c} G(\omega ,E)\approx G(\omega ). \end{array}$$

Similarly, the mean DoS ρ(E) is almost independent of *E* and can be replaced by $\rho_{0}\equiv \rho \left(0\right)=1$. Integrating over $E+E^{\prime }$ at a fixed *ω* we obtain:

[31] $$\begin{array}{c} \Sigma^{2}=\int_{-\Delta }^{\Delta }\left(\Delta -|\omega |\right)\;R\left(\omega \right)\;d\omega , \end{array}$$

where

[32] $$\begin{array}{c} R(\omega )=G(\omega )-1. \end{array}$$

It immediately follows from Eq. **31** that:

[33] $$\begin{array}{c} \chi \left(\Delta \right)=\frac{d\Sigma^{2}}{d\Delta }=\int_{-\Delta }^{\Delta }R\left(\omega \right)\;d\omega . \end{array}$$

Notice that R(ω) contains a δ(ω) part coming from the term in the sum with α=β in Eq. **26**.

Thus we have

[34] $$\begin{array}{c} \chi \left(\Delta \right)=1+2\int_{+0}^{\Delta }R\left(\omega \right)\;d\omega , \end{array}$$

where we assume that all energies are measured in units of *δ* and R(ω) is an even function. Apart from this last assumption, Eq. **34** is absolutely general.

The critical R(ω) is characterized by the limiting $R_{\infty }\left(\omega \right)=\;\lim_{N\to \infty }R\left(\omega \right)$. It decreases fast enough as ω→∞, so that the integral in Eq. **34** is convergent at Δ→∞ and negative due to level repulsion. In contrast, for an insulator $R_{\infty }\left(\omega \right)\equiv 0$ at N→∞. For a metal, R(ω) is described by the Wigner-Dyson theory for *ω* much smaller than the Thouless energy $E_{\mathrm{Th}}$, which, in units of *δ*, tends to infinity $E_{\mathrm{Th}}/\delta \equiv g\left(N\right)\to \infty$ as N→∞.

If the energy range of a filter function is *N*-independent, it will be infinite in the limit N→∞ when measured in units of δ∼1/N. Assuming this limit is taken first and the integral in Eq. **34** is convergent, we conclude that the critical spectral compressibility tends to a constant 0<χ<1 at Δ→∞.

[35] $$\begin{array}{c} \chi =\lim_{\Delta \to \infty }\lim_{N\to \infty }\chi \left(\Delta \right)=1+2\int_{+0}^{\infty }R_{\infty }\left(\omega \right)d\omega \end{array}$$

For localized states Eq. **35** gives χ=1, and for the ergodic delocalized ones χ=0, since for all symmetry classes of Wigner-Dyson theory $\int_{+0}^{\infty }\lim_{N\to \infty }R\left(\omega \right)=-1/2$.

Now return back to Eq. **25** and cast it as

[36] $$\begin{array}{c} K\left(\tau \right)=FT\left\{\frac{1}{Z}\int dE\;\eta \left(E\right)\eta \left(E-\omega \right)\;\left[R\left(\omega \right)+1\right]\right\}. \end{array}$$

For a Gaussian filter ∫dE η(E)η(E−ω) is a Gaussian function of the width which is $\sqrt{2}$ larger than that of η(E). If this width is *N*-independent in conventional energy units, it will be infinite when measured in units of *δ* in the large-*N* limit. At the same time, for critical states the range of the correlation function G(ω) which makes the dominant contribution to Eq. **35**, is finite. This separation of scales allows to replace:

[37] $$\begin{array}{c} \frac{1}{Z}\int dE\eta \left(E\right)\eta \left(E-\omega \right)\to \frac{1}{Z}\int dE{\left[\eta \left(E\right)\right]}^{2}=const. \end{array}$$

One can always take the normalization *Z* such that this constant is equal to 1. Taking also into account that the Fourier transform of a constant is a δ(τ)- function we conclude from Eq. **36** that in the limit N→∞ taken first and a proper choice of *Z* one has

[38] $$\begin{array}{c} \chi =\lim_{\tau \to +0}\lim_{N\to \infty }K\left(\tau \right)=\lim_{\Delta \to \infty }\lim_{N\to \infty }\chi \left(\Delta \right). \end{array}$$

For a finite *N* the spectral form factor K(τ) is a nontrivial function of *τ*. It increases sharply at $\tau ≲{\left(\eta_{0}\;N\right)}^{-1}$, where $\eta_{0}$ is the width of the filter function η(E). The sharp increase of K(τ) at a small *τ* seen in Fig. 1*A* is given by the Fourier transform of the term $Z^{-1}\int dE\;\eta \left(E\right)\eta \left(E-\omega \right)$ in Eq. **36** which is nothing but the broadened δ(τ) function discussed above. For the values of *τ* in the interval $\delta /E_{\mathrm{Th}}≲\tau ≲1$ the function R(ω) is described by the Wigner-Dyson theory: $R\left(\omega \right)\propto -1/\omega^{2}$, while K(τ)∝τ grows linearly and approaches the universal limit K(τ≫1)=1 due to the δ(ω) function in R(ω). In between, for ${\left(\eta_{0}\;N\right)}^{-1}\ll \tau \ll \delta /E_{\mathrm{Th}}$, there is a plateau for the critical K(τ) which height is given by Eq. **35**.

### Derivation of Eqs. **16** and Eq. **17**.

The derivation of Eq. **16** for the unitary symmetry class is based on Eq. **35** and the global DoS correlation function computed in ref. 24,

[39] $$\begin{array}{c} R\left(\omega \right)=\delta \left(\omega \right)-F{\left(\omega \right)}^{2}, \end{array}$$

where the limit N→∞ is already taken and

[40] $$\begin{array}{c} F\left(\omega \right)=\frac{1}{4b}\frac{\sin(\pi \omega )}{\sinh\;\left(\frac{\pi \omega }{4b}\right)}. \end{array}$$

Eq. **40** was derived using the nonlinear sigma model (NLSM) perturbative approach of ref. 40 extended to the case of the critical PLRB model in ref. 24.

By derivation, Eq. **40** is valid for ω≫1 (in units of *δ*) and for b≫1 when the NLSM is justified. The correlation function R(ω) takes a well-known form in the limit b→∞ when it exactly coincides with the Wigner-Dyson global DoS correlation function (1) for all values of *ω* down to zero. Thus the perturbative derivation, which is under control only for ω≫1, appears to give an exact result for R(ω) in the Wigner-Dyson unitary case. This fact is known as a “perturbative exactness.”

One could hope that Eq. **40** has the same property of perturbative exactness and is also valid for all *ω*, provided that b≫1. And, indeed, it was shown in ref. 25 that it gives a correct leading term at ω/b≪1 found in ref. 58. Thus Eq. **40** gives a correct leading behavior both at ω≫1 and in ω≪b. Since these two domains overlap at b≫ω≫1, Eq. **40** should give a correct asymptotic behavior for all *ω*, provided that b≫1.

Notice also that the DoS correlation function, Eq. **39**, has a structure of the Wigner-Dyson theory $R\left(\omega >0\right)=-F{\left(\omega \right)}^{2}$ for the unitary symmetry class, albeit with the modified kernel F(ω). As a matter of fact, this kernel corresponds to the one for the noninteracting fermions in 1*d* at a finite temperature,

[41] $$\begin{array}{c} T=\frac{1}{4b}, \end{array}$$

while the standard Wigner-Dyson kernel corresponds to zero temperature.

What remains to be done to find *χ* in the unitary ensemble is to integrate R(ω) as in Eq. **35**. So we obtain Eq. **16**.

As was explained above, the correctness of the result is guaranteed only for b≫1. However, Eq. **16** describes *χ* as a function of *b* extremely well for all values of parameter *b* in the unitary PLRB model, see Figs. 3*B* and Fig. 4*A*.

In the standard Wigner-Dyson theory of orthogonal symmetry, the relation between R(ω) at N→∞ and F(ω) at b→∞ is more complicated (1),

[42] $$\begin{array}{c} R\left(\omega >0\right)=-F{\left(\omega \right)}^{2}-\frac{\mathrm{dF}}{d\omega }\int_{\omega }^{\infty }F\left(x\right)dx,\;F\left(-\omega \right)=F\left(\omega \right). \end{array}$$

One may compute R(ω) using the same kernel, Eqs. **40** and **42** and check if it gives a correct leading term in R(ω) at small ω<1. It was done in ref. 25 with the affirmative answer. This demonstrates that the status of Eq. **42** with the kernel, Eq. **40**, is the same as for the unitary ensemble: It is valid for all *ω* in the limit b≫1.

Let us compute *χ* from Eq. **35** using R(ω) found as described above. It is convenient first to integrate by parts over *ω* and then integrate over *x* in Eq. **42**. So we obtain:

[43] $$\begin{array}{c} 2\int_{+0}^{\infty }R\left(\omega \right)\;d\omega =2\int_{0}^{\infty }F\left(x\right)dx-4\int_{0}^{\infty }F^{2}\left(x\right)dx. \end{array}$$

Then, using

[44] $$\begin{array}{c} \int_{0}^{\infty }F\left(x\right)dx=\frac{1}{2}\tanh\left(2\pi b\right), \end{array}$$

[45] $$\begin{array}{c} \int_{0}^{\infty }F^{2}\left(x\right)dx=-\frac{1}{8\pi b}+\frac{1}{2}\coth\left(4\pi b\right), \end{array}$$

one obtains Eq. **17** from Eq. **35**.

By derivation, the status of Eq. **17** should be the same as Eq. **16**: It should be valid asymptotically at b≫1. And, indeed, Fig. 4 shows that this is the case. However, for intermediate values of *b* the agreement with numerical results is not as good as for the unitary ensemble.

## Data Availability

The displayed data of this work is available in an openly accessible repository (59).

## Footnotes

^*^There was a misprint in ref. 39. It is corrected in Eq. **14**.
